# Implementing the World Health Organization Guidelines for Rheumatic Heart Disease in Highly Endemic Settings: Balancing Evidence with Reality

**DOI:** 10.5334/gh.1527

**Published:** 2026-02-16

**Authors:** Sulafa K. M. Ali

**Affiliations:** 1 Department of Clinical Sciences, College of Medicine, University of Sharjah, United Arab Emirates

**Keywords:** rheumatic heart disease, world health organization guidelines

## Abstract

In 2024, the World Health Organization (WHO) released rheumatic heart disease (RHD) guidelines; however, implementation in highly endemic countries remains challenging. The severe nature of RHD in such areas necessitates adopting pragmatic approaches to improve diagnostic yield. The WHO continues to recommend the Jones Criteria for the diagnosis of acute rheumatic fever, even though these criteria have been shown to miss up to 10% of cases. Using simplified clinical criteria is expected to have better sensitivity at the level of frontline health workers. The WHO recommendation to use handheld echocardiography for diagnosis of RHD is a crucial asset for secondary and tertiary prevention of RHD; however, implementation of this strategy might be hampered by limited availability and the need for expert training. While benzathine penicillin G remains the cornerstone of RHD treatment, as recommended by the WHO, persistent issues related to its availability and proper administration techniques need to be addressed.

## Introduction

Rheumatic heart disease (RHD) is a major cause of acquired heart disease among young people, particularly in low- and middle-income countries (LMICs), leading to significant complications including premature cardiac death (1).

According to the latest Global Burden of Disease data from 2021, RHD affects 55 million people, with an annual increase of 1.7%. This rise could be attributed to the increased availability of echocardiography (echo), which has led to the detection of many mild and subclinical cases of RHD. The burden of RHD is largely borne by marginalized nations where factors such overcrowding, malnutrition and poor access to health care are prevalent (2).

The prevalence of RHD has declined in developed countries, largely attributed to improvements in sanitation and access to health care. This implies that the disease is preventable if socioeconomic determinants of health are effectively addressed (3).

Although the global trends in RHD mortality show improvement, the persistent high burden in some areas underscores the disparities in health care systems and socioeconomic determinants of health. Highly endemic areas include sub-Saharan Africa, Central and South Asia and the South Pacific. The highest burden of RHD was found to be in African countries, where the age-standardized incidence rate is 93/100,000, nearly double the average global rate of 50/100,000. Other WHO regions have a rate of 12–53/100,000. Within the African regions, there are significant disparities in the incidence of RHD: Northern Africa reports an incidence of 50/100,000, in sharp contrast with Southern Africa, which reports incidence as high as 129/100,000 (4,5).

It is important to note that within countries, RHD tends to cluster in certain geographical regions, typically impacting less advantaged, marginalized populations (6).

The high endemicity and mortality in these areas are due to a combination of socioeconomic and health system factors coupled with political instability. War and displacement often disrupt efforts to control the disease. An example of such disruption is the collapse of the control of RHD in Sudan following the 2023 war. Prior to the war, a multisectoral program had been partially implemented and country guidelines, endorsed by the regional WHO and the local government, were established; the war severely hampered their implementation (7,8).

While the latest WHO guidelines (9) are based on scientifically valid evidence, the overwhelming majority of RHD cases occur in areas where obtaining high-quality research evidence is often challenging, a painful reality that confronts health systems in such countries.

## WHO Guidelines: Balancing Evidence with Reality

Implementation of the 2024 WHO recommendations in highly endemic countries has faced significant obstacles. In the following sections we will discuss the benefits and limitations of the WHO guidelines.

### 1. Diagnosis and treatment of group A streptococcal (GAS) pharyngitis

The guidelines acknowledged the poor access to diagnostic tests for GAS pharyngitis, such as point-of-care rapid antigen tests and microbiological culture. Thus, it is recommended that antibiotic treatment be initiated in highly endemic countries based solely on clinical suspicion. This recommendation is particularly important in low-resource settings as antibiotics not only reduce the prevalence of acute rheumatic fever (ARF) but also decrease suppurative complications of GAS such as quinsy. There has been some progress with diagnosis of GAS pharyngitis using molecular point-of-care testing in Australia. The test has a documented sensitivity of 100% and specificity of 79%, with reported positive predictive values of 49% and negative predictive values of 100% compared with throat culture (10). A challenge remains in ensuring that such investigations are available and affordable in limited-resource settings. The antibiotic of choice is penicillin; an intramuscular single dose of benzathine penicillin G (BPG) is preferred over oral antibiotics because of better patient compliance. Oral antibiotics need to be taken for 10 days; this long duration of treatment often results in poor patient compliance. The limitations of BPG are discussed in section 4.

### 2. Diagnosis of ARF

In the absence of a definitive diagnostic test for ARF, the current WHO guidelines recommended that the Jones Criteria (JC), originally established in 1944 and modified in 2015, should continue as the reference for diagnosis of ARF (11).

These criteria have been the gold standard diagnostic method for over 80 years; however, the disease remains significantly underdiagnosed, particularly in primary health care settings (12,13). The main limitations of early diagnosis of ARF using the JC include the following:

1. The JC include five major and four minor features in addition to obtaining evidence of GAS infection. This extensive number of items is difficult to apply effectively in primary health care settings.
2. The JC require laboratory testing including total white blood cell count, erythrocyte sedimentation rate and C reactive protein, in addition to antistreptolysin O titer to confirm GAS infection. Additionally, electrocardiography and echo are required to assess cardiac involvement. These investigations are usually limited in remote primary health care centers where the initial ARF episode typically occurs.
3. The transient nature of arthritis may lead to late presentation to secondary care settings; therefore, acute arthritis alone as an early symptom of ARF needs to be over-emphasized in primary care settings.
4. The clinical features of ARF overlap with those of common infections, particularly with malaria and some viral infections. Malaria is often treated empirically and, as the fever subsides and arthritis resolves, ARF could be overlooked. Coincidence of malaria and ARF has been documented in Sudan as well as in Uganda (14,15).
5. The fact that carditis can be subclinical indicates that the first episode could be missed due to the absence of other major criteria. The use of handheld echo (see below) could help in revealing the diagnosis.

These factors lead to difficulty in diagnosing the first episode of ARF, especially in endemic areas where frontline health workers have limited clinical and laboratory facilities. The disease’s complexity and similarity with other endemic diseases, coupled with poor access to investigation facilities, are important factors that hamper the implementation of the JC. In such areas, obtaining consultations with physicians in secondary or tertiary centers often requires several months, due to significant barriers including the prohibitive costs of travel and the long distances to referral hospitals.

It has been documented that, in febrile children living in endemic areas who do not have any clinical signs of ARF, screening by handheld echo can unmask subclinical carditis as the only major criterion for ARF (14). In this study, which included 400 febrile children, echo improved the diagnosis of ARF by 11%. The findings of this study strongly support the use of echo screening in primary health care settings. Considering the limitations of the JC, a diagnostic test for ARF is urgently needed. Two biomarker discovery studies are currently underway: the ‘Searching for a Technology-Driven Acute Rheumatic Fever Test’ (START) study in Australia and New Zealand aims to detect biomarkers that distinguish ARF cases using serology from patients and controls (16).

**Figure 1:**
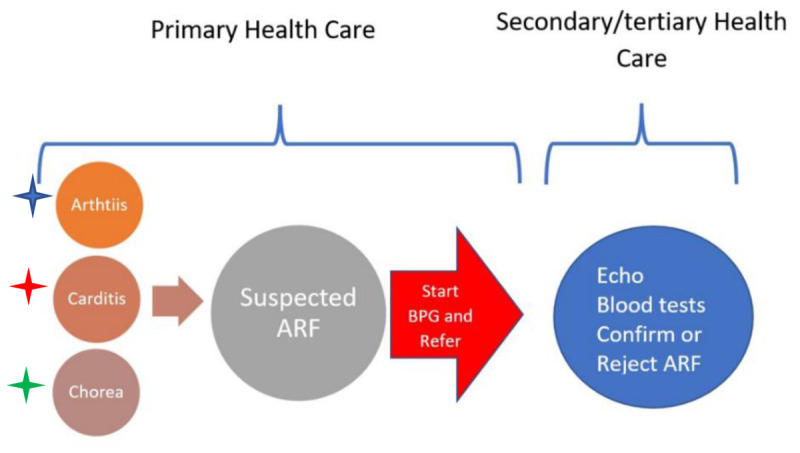
Simple algorithm for diagnosis of ARF at primary health care centers. 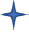
 Arthritis: Acute migratory polyarthritis or polyarthralgia. Monoarthritis and monoarthralgia need to be referred without prescribing BPG. 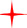
 Carditis: Murmurs of mitral and/or aortic valve disease with or without heart failure. 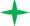
 Chorea alone is enough to diagnose ARF.

The other is the ARF Diagnostic Collaborative Network, which includes patients from different continents and aims to detect an ARF biomarker (17). Pending discovery of a reliable biomarker for ARF, a wider diagnostic framework is needed to improve ARF detection in endemic areas. Using a simplified pragmatic algorithm that could easily be interpreted by frontline health workers could improve diagnosis and management at the primary health care level (18) (Figure 1). The addition of handheld echo in primary health care settings is expected to improve the performance of this algorithm. Investing in training of primary health care personnel in handheld echo could achieve many objectives, including RHD control, as well as screening and management of other medical and obstetric conditions.

Although the evidence for using a simplified approach for ARF diagnosis has not been supportive of WHO recommendation, several studies have reported poor performance of the JC for ARF diagnosis. A systematic review which included three studies found that a simplified algorithm using clinical data at community level was 66% sensitive and 68% specific, and had worse performance than the JC, which performed at 84 and 87%, respectively. However, the JC without echo led to a loss of sensitivity down to 79%. It is estimated that 2.5–5% of patients who do not meet the JC progress to RHD. This finding supports using a simplified clinical algorithm, particularly in highly endemic areas (19).

The algorithm shown in Figure 1 has been approved by the Sudan RHD guideline committee and was endorsed by the local Ministry of Health and the RHD Committee of the East Mediterranean Region of the WHO in 2023 (8). The main benefit of this approach is the prompt initiation of antibiotic prophylaxis covering the period between presentation and referral to a higher center.

### 3. Echo Screening

The WHO guidelines support the use of point-of-care echo both for diagnosis and for screening of children, young adults and pregnant women when standard echo is not available. This recommendation is in accordance with the evidence for the reliability of handheld echo performed by expert as well as nonexpert operators. In addition, the availability of evidence-based echo criteria from the World Heart Federation, updated in 2023, provides an important reference for echo diagnosis of RHD (20). This recommendation is expected to expand the role of echo screening in highly endemic areas to be a tool for disease control through early detection and timely institution of secondary prophylaxis. However, many limitations are expected, including the availability of echo machines, training of health workers in rural areas and verification of the echo results by specialized personnel. On the other hand, ultrasound technology is rapidly evolving, with availability of low-cost machines that could have a central role in control programs if expert supervision is provided. Technical support and continuous governance and monitoring are needed, which could be a major limitation to implementing this strategy in low-resource settings. Additionally, the cost-effectiveness of this policy needs to be carefully evaluated, particularly given the increased workload for referral centers, which already contend with limited availability of standard echo machines and expert staff.

### 4. Antibiotic prophylaxis for the prevention of recurrent rheumatic fever

The current guidelines recommend giving prophylactic antibiotics, namely BPG every three to four weeks, to patients with RHD, including those with minimal echo criteria. Using secondary prophylaxis for patients with subclinical RHD discovered on screening programs is expected to improve the control of RHD in endemic areas. The supply of BPG as well as oral penicillin V has been disrupted in many countries; this situation needs urgent intervention by the WHO at the level of pharmaceutical manufacturers (21).

**Figure 2:**
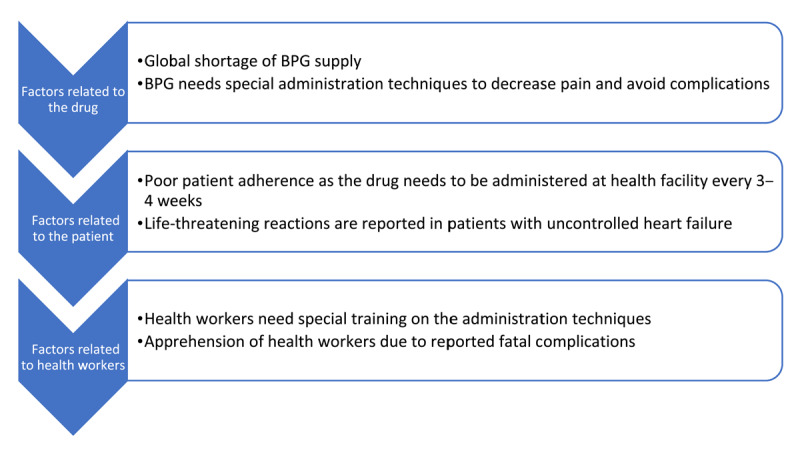
Factors limiting the use of BPG.

The administration of BPG has been challenged by factors related to the drug, and to patients and health workers, as shown in Figure 2.

Cases of sudden death following BPG have been reported in many countries and typically occur immediately following the injection without signs of anaphylaxis. In a recent meta-analysis the incidence of severe reactions following BPG was found to be 9.7 per 10,000 and 1.1 per 10,000 injections. Fatality was reported to be 0.05%, and all those who died had severe RHD (22). Although not fully understood, the cause of sudden death was thought to be attributable to an arrhythmic or vasovagal event, which occurred mostly in those with severe valve disease. Although such events are rare, they can cause significant anxiety among families and health workers, leading to disruption of control programs. This problem has been addressed by a statement from the American Heart Association recommending avoidance of BPG in those with severe valve disease (23). However, this recommendation raised concerns in highly endemic countries, as the rate of severe RHD is high, reaching up to 65% of cases seen in referral hospitals (24). Considering this level of severity, the strategy of avoiding BPG in those with severe single valve disease poses the risk of multiple valve affection. In addition, health workers will be discouraged from administering the injection and families’ adherence will be adversely affected. The WHO recommendations to use BPG need to be coupled with efforts to improve the supply and to train health workers. Efforts to develop better formulations of long-acting antibiotics are ongoing. Supply of oral penicillin needs to be improved, as it will be an alternative to BPG for patients at high risk of fatal reactions.

## Research Gaps and Future Perspectives

Reliable and affordable diagnostic tests for GAS as well as for ARF are urgently needed. Investigations for such tests need to include RHD-endemic countries from diverse geographical areas to ensure their reliability. A simplified algorithm for ARF that utilizes handheld echo needs to be tested at primary care level. There is a need to investigate the causes and risk factors associated with fatal reactions related to BPG. Striving to discover an antibiotic substitute with better administration techniques and safety profile remains one of the strategic needs for RHD control.

## Conclusion

The WHO recommendations incorporated important additions, including echo screening as a key strategy for RHD control. However, implementing ARF diagnosis in endemic areas needs adapted guidelines that consider the severity of the disease; in this article, a pragmatic approach is suggested. Limitations of BPG supply and challenges with its administration need to be addressed to ensure the effectiveness of primary and secondary prophylaxis.
